# Robotic transthoracic esophagectomy

**DOI:** 10.1186/s12893-015-0024-2

**Published:** 2015-04-23

**Authors:** Shailesh Puntambekar, Rahul Kenawadekar, Sanjay Kumar, Saurabh Joshi, Geetanjali Agarwal, Sunil Reddy, Jainul Mallik

**Affiliations:** Galaxy Care Laparoscopy Institute, Opposite Garware College, 25-A, Ayurvedic Rasashala Premises, Karve Road, Pune, Maharashtra 411004 India; Department of Surgery, JNMC, KLE University, Belgaum, India

## Abstract

**Background:**

We have initially published our experience with the robotic transthoracic esophagectomy in 32 patients from a single institute. The present paper is the extension of our experience with robotic system and to best of our knowledge this represents the largest series of robotic transthoracic esophagectomy worldwide. The objective of this study was to investigate the feasibility of the robotic transthoracic esophagectomy for esophageal cancer in a series of patients from a single institute.

**Methods:**

A retrospective review of medical records was conducted for 83 esophageal cancer patients who underwent robotic esophagectomy at our institute from December 2009 to December 2012. All patients underwent a thorough clinical examination and pre-operative investigations. All patients underwent robotic esophageal mobilization. En-bloc dissection with lymphadenectomy was performed in all cases with preservation of Azygous vein. Relevant data were gathered from medical records.

**Results:**

The study population comprised of 50 men and 33 women with mean age of 59.18 years. The mean operative time was 204.94 mins (range 180 to 300). The mean blood loss was 86.75 ml (range 50 to 200). The mean number of lymph node yield was 18. 36 (range 13 to 24). None of the patient required conversion. The mean ICU stay and hospital stay was 1 day (range 1 to 3) and 10.37 days (range 10 to 13), respectively. A total of 16 (19.28%) complication were reported in these patents. Commonly reported complication included dysphagia, pleural effusion and anastomotic leak. No treatment related mortality was observed. After a median follow-up period of 10 months, 66 patients (79.52%) survived with disease free stage.

**Conclusions:**

We found robot-assisted thoracoscopic esophagectomy feasible in cases of esophageal cancer. The procedure allowed precise en-bloc dissection with lymphadenectomy in mediastinum with reduced operative time, blood loss and complications.

**Electronic supplementary material:**

The online version of this article (doi:10.1186/s12893-015-0024-2) contains supplementary material, which is available to authorized users.

## Background

The GLOBOCAN 2008 cancer fact sheet described esophageal cancer as the eighth most common cancer worldwide. There were 482,300 new cases of esophageal cancer with 406,800 estimated deaths worldwide in year 2008 [1]. For esophageal cancer, esophagectomy remains the cornerstone of the treatment with curative intent [2]. However, surgical resection of the esophageal cancer is a challenging dissection compared to other gastrointestinal cancers mainly due to anatomical difficulties. Additionally, surgeries for esophageal cancer are frequently associated with high rates of cardiopulmonary morbidity and mortality [3,4]. Minimally invasive esophagectomy (MIE) is being used increasingly with aim to reduce the surgical trauma and thereby reducing morbidity and mortality [4-6]. The available literature data shows other advantages of MIE including decrease in operative time, blood loss, post-operative complications and hospital stay with comparable oncological clearance [4-6]. However, steep learning curve associated with MIE has been a challenge [7,8].

Robotic surgery has generated considerable excitement and interest in various oncological surgeries including esophageal surgery. Robot-assisted surgery can accelerate the learning curve of MIE with the help of magnified three-dimensional view, improved articulation of instruments with seven degree of freedom, improved dexterity and enhanced ergonomics [8-11]. Robotic assisted surgery can help the surgeon in precise dissection of the structures in the mediastinum which otherwise would have been challenging via conventional MIE. However, in spite of various advantages of robotic esophagectomy, it still remains in the early phase of acceptance.

We have initially published our experience with the robotic transthoracic esophagectomy in 32 patients from a single institute [11]. The present paper is the extension of our experience with robotic system and to best of our knowledge this represents the largest series of robotic transthoracic esophagectomy worldwide.

## Methods

### Patients

We retrospectively reviewed the medical records of 83 esophageal cancer patients who underwent robotic assisted esophagectomy at Galaxy Care Laparoscopy institute from December 2009 to December 2012. The review was conducted on the basis of prospectively recorded data from a computerized database. Care Hospitals Institutional review board and ethics committee approved the study. Each patient gave consent for conversion to either thoracoscopic or open surgery just in case of complications. Each patient’s consent was also taken for use of video footage of their surgery for academic and research purposes (including specific written consent from the patient whose edited operative video is accompanying this manuscript). All patients underwent a thorough clinical examination and pre-operative investigations including routine hematogram, biochemical investigations, pulmonary function tests, chest radiograph, ECG, 2D Echo, contrast enhanced CT scan of thorax and abdomen, upper GI endoscopy with biopsy and barium swallow with stomach plates. Endoscopic ultrasound staging was done to identify the stage of the tumor.

Patients with histologically proven squamous cell carcinoma, adenocarcinoma or dysplasia as well as other resectable forms of tumors were the candidate for the surgery. The operability criteria were clearly defined as per NCCN guidelines. American Society of Anesthesiologists (ASA) class I to III was regarded appropriate for the procedure. Patients with cervical esophageal cancer were not considered suitable for the procedure.

All the patients underwent robotic esophageal mobilization. The entire thoracic esophagus along with paraesophageal, subcarinal, paratracheal and bronchial lymph nodes were removed en-bloc. Azygos vein was preserved in all cases. The resected specimen was examined by an experienced pathologist. Pre-operative investigations, clinical data, operative details, pathological details and post-operative data were gathered from medical records.

### Operative technique

**Figure 1:**
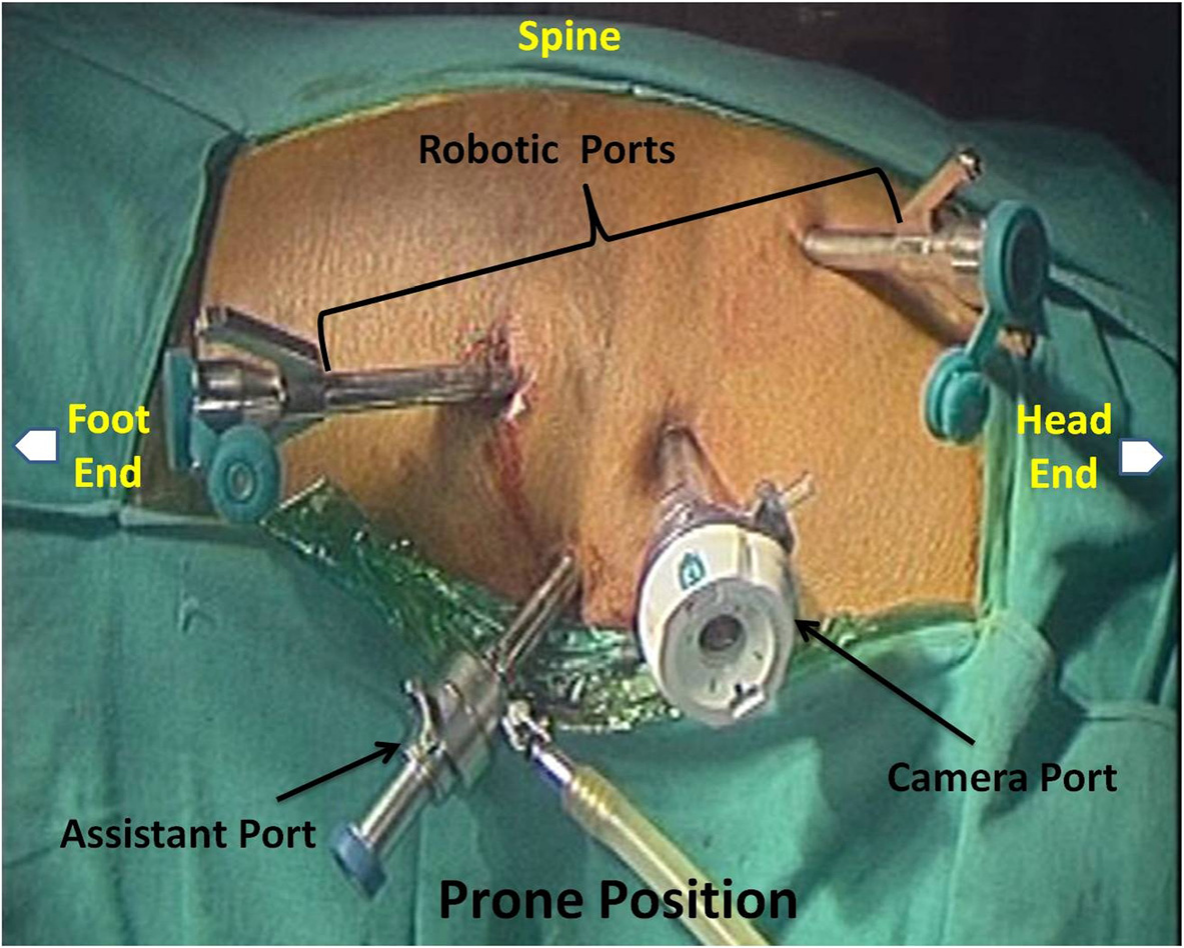
Patient and port positioning.

The patient was placed in the prone position on an operative sandbag. The robotic cart was situated to the left side of the patient. The operative trocars for the robot (one 12- mm port for the camera and two 8-mm ports for the arms) were placed (Figure 1). The first port was inserted 1 finger-breadth below and posterior to inferior angle of scapula in the 5th or 6th intercostal space. Two 8-mm trocars were positioned under direct thoracoscopic vision in a vertical line at a distance of 5 cm and in triangulation with the camera port in the third and eighth intercostal spaces, respectively. One 10-mm port for the assistant was placed between the left working port and the camera port. This was used for suction and clip application. Pneumoinsufflation was created at a pressure of 7 mm Hg. (Additional file 1).

With the patient in a prone position, the esophagus falls anteriorly out of its normal position, which creates natural tension and simplifies dissection. We used Maryland bipolar forceps in left arm and hot shears (scissors with monopolar current) in right arm of the robot. The procedure began with the incision of the visceral pleura between the esophagus and the lung just inferior to the azygos vein. This helped in keeping the esophagus attached to the pleura on the aortic side. More than 3 fourths of the circumference of the esophagus was mobilized in this way from the cranial to the caudal direction. The plane of dissection was outside the vagus. The posterior large direct aortic branches were then clipped, and the small branches were cauterized with bipolar forceps. This completed the mobilization of the esophagus all around. The caudal limit of the dissection was the hiatus. The same dissection was continued in the supra-azygos region. The vagal fibers going to the bronchus were preserved. The azygos vein was preserved. Complete mobilization of the esophagus was achieved. The specimen also included the lower and middle mediastinal, subcarinal, and right paratracheal nodes. The thoracic duct was identified and clipped in all cases.

#### Stomach mobilization

Stomach mobilization was done laparoscopically. All the nodes along the left gastric, paraesophageal, splenic and along the hepatic artery were removed. The stomach was mobilized on the right gastro epiploic and right gastric artery. The stomach tube was prepared extracorporially by taking 5 cm incision in the midline.

#### Cervical incision

Left supraclavicular 3 cm incision was taken. The two heads of the sternomastoid were separated and the esophagus was pulled up to the wound. The esophagus was cut and the NG tube was attached to the distal end. The specimen was removed abdominally. The stomach tube was attached to the NG tube and was pulled to the neck and hand sewn esophageogastric anastamoses was done. Feeding jejunostomy was done in all the patients.

### Post-operative course

The patients were kept in ICU for minimum one day after which shifted to respective ward. The continuous monitoring of vital parameters was done further for next 48 hrs and then every day for the next 4 days. The chest radiograph and hematogram was done every day for 2 days and then were repeated only when the patient had respiratory signs, fever, or any other complaints. Patients were made ambulatory after 24 hrs and jejunostomy tube feeding was started after 48 hrs of surgery. The leak test was performed on the 7^th^ day of the surgery and oral feeding was started accordingly.

The patients with nodal involvement were subjected to chemotherapy or radiotherapy or both depending on number of node positivity and histology of tumor. The patients were followed 3 monthly for clinical checkup, chest radiograph and ultrasonography for the period of one year and than six monthly for a period of two years. If any suspicion was raised PET scan was advised.

## Results

The study population consisted of 83 patients, 50 men (60.24%) and 33 women (39.76%). The mean age at surgery was 59.18 years (range, 30 to 87 years). Most commonly involved site (50 cases, 60.24%) was lower third of the esophagus. The predominant histology (67 cases, 80.72%) was squamous cell carcinoma. A total of 21.69% of patients presented at least one comorbid condition, with hypertension and diabetes observed as most common comorbidities. Information on patient demographics is presented in Table 1.

**Table 1:** **Clinico-demographic characteristics of the patients with esophageal cancer**

***Variable***	***N (%)***
**Gender**	
Male	50 (60.24)
Female	33 (39.76)
**Age distribution**	
21 – 30	1 (1.20)
31 – 40	5 (6.02)
41 – 50	15 (18.07)
51 – 60	22 (26.51)
61 – 70	28 (33.73)
71 – 80	9 (10.84)
81 – 90	3 (3.61)
**Histology**	
Squamous cell carcinoma	67 (80.72)
Adenocarcinoma	12 (14.46)
Dysplasia	2 (2.41)
Gastrointestinal stromal tumor	1 (1.20)
Lymphoma	1 (1.20)
**Site of cancer**	
Middle third	50 (60.24)
Lower third	20 (24.10)
Gastroesophageal junction	13 (15.66)
**TNM stage of tumor**	
High-grade dysplasia	2 (2.41)
I	8 (9.64)
II	64 (77.11)
III	9 (10.84)
**ASA grading**	
I	2 (2.41)
II	60 (72.29)
III	19 (22.89)
IV	2 (2.41)
**Preoperative morbidity**	
Diabetes mellitus	11 (13.25)
Bronchial asthma	3 (3.61)
Hypertension	15 (18.07)
Stroke	1 (1.20)
Ischemic heart disease	2 (2.41)
Hemiplagia	1 (1.20)

The mean operative time was 204.94 minutes (range 180 to 300). The size of the tumor did not significantly affect operative times. The mean blood loss was 86.75 ml (range 50 to 200). The mean number of lymph node yield was 18. 36 (range 13 to 24). Only two patients showed positive circumferential margin. None of the patient required conversion to thoracoscopic or open surgery. The mean ICU stay was 1 day (range 1 to 3) and the mean hospital stay was 10.37 days (range 10 to 13). Table 2 represents operative and post-operative outcomes.

**Table 2:** **Operative outcomes following the robotic transthoracic esophagectomy**

***Variable***	***Mean (range) or no. (%)***
Total operative time (mins)	204.94 (180–300)
Docking time (mins)	9.06 (5–30)
Undocking time (mins)	5 (2–10)
Time for esophageal mobilization (mins)	104.08 (80–170)
Estimated blood loss (ml)	86.75 (50–200)
Lymphnode yield (no.)	18.36 (13–24)
ICU stay (days)	1 (1–3)
Hospital stay (days)	10.37 (10–13)
Nil by mouth (days)	9.40 (8–12)
Conversion (no.)	Nil
Margin positivity (no.)	2 (2.41)
Adjuvant chemotherapy (no.)	15 (18.07)
***Complications (no.)***	
Dysphagia for solids	6 (7.23)
Pleural effusion	3 (3.61)
Aspiration pneumonia	1 (1.20)
Recurrent palsy	2 (2.41)
Anastomotic leak	3 (3.61)
Chyle leak	1 (1.20)
Port site metastasis	1 (1.20)
Surgical site infection	1 (1.20)
Sepsis	1 (1.20)

**Figure 2:**
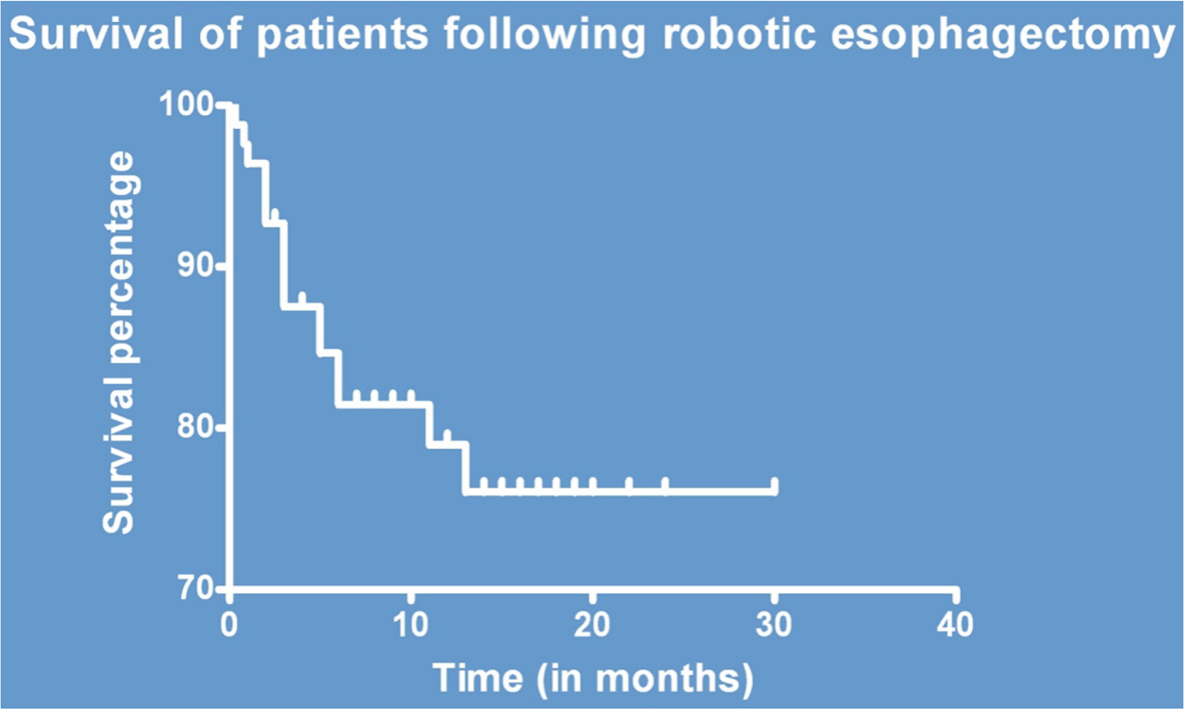
Disease free survival following robotic transthoracic esophagectomy (Kaplan Meier Plot).

Postoperative morbidity occurred in 16 cases (19.28%). A total 19 of incidents were reported in these cases. Dysphagia was the most commonly observed event (6 cases, 7.23%) followed by pleural effusion (3 cases, 3.61%). Anastomotic leak was reported in 3 cases and chyle leak was observed in one patient. There were only two incidences of recurrent palsy. One patient developed port site metastasis which was treated with excision. No treatment related mortality was observed. At the end of the study period, 66 patients (79.52%) were alive in disease free stage at the median follow-up period of 10 months. One patient was lost to follow up. Figure 2 shows the Kaplan Meier survival analysis.

## Discussion

To the best of our knowledge, this is the largest study on robotic transthoracic esophagectomy till date which represents the comprehensive experience from our institute. As the available data in the literature on this advancement is scarce, we feel that the present study perhaps will provide a better idea on the ‘real world’ scenario.

Esophagectomy for esophageal carcinoma is a technically challenging procedure associated with relatively high mortality and morbidity [7,12-14]. To reduce the morbidity as a result of surgical trauma from open procedures, minimal invasive procedures were introduced in the recent past. There are published reports that favored the use of MIE due to advantages of shorter operative time; reduced blood loss and shorter hospital stay [7,12,15-17]. Nevertheless, the conventional MIE methods are limited by the technical difficulties. Mainly, the use of long instruments with limited degree of freedom and two-dimensional view can become hindrance for optimal dissection [18,19].

The more recent introduction of robotic systems in surgical oncology has answered the limitations of MIE. Compared to the traditional minimally invasive procedures, robotic-assisted surgery offers several potential advantages. The improved visualization with the magnified three-dimensional view is of particular benefit that allows a precise and atraumatic dissection of the peri-esophageal tissue along the vital structures, such as pulmonary vein, trachea, thoracic duct, aorta and vagus nerve [9,20]. More importantly, the magnified view of the surgical field can assist in a more extensive dissection of the lymph nodes. The lymphatic spread of esophageal cancer is generally irregular due to submucosal lymphatic drainage system. The radical resection of esophagus with surrounding lymph nodes offers the best possible cure. With the use of robotic arms, we were able to achieve the mean lymph node yield of 18.36 (range 13 to 24). A study from van Hillegersberg R et al. harvested a median of 20 lymph nodes (range, 9–30) through robot-assisted thoracoscopic esophagectomy [9]. Cerfolio et al. reported median number of 20 through the same approach [21]. Galvani et al. achieved mean lymph nodes yield of 12 (range 7 to 27) using robot-assisted transhiatal esophagectomy [22]. The same approach was utilized by Dunn et al., who harvested a median of 20 lymph nodes (range, 3–38) [23]. Sarkaria et al. reported median number of 20 lymph nodes (range, 10–49) using combined thoracoscopic and laparoscopic robotic-assisted approach [24]. We believe that the transthoracoscopic approach offers an outstanding access to the mediastinum and thereby allows an extended lymphadenectomy. When performing the procedure through the transhiatal approach, these potential metastatic lymph nodes might be leftover in situ [25]. In fact, the R0 resection was achieved in 97.59% of our study population. Few other case series using the similar approach showed R0 resection rate varying from 76% to 100% [21,26,27].

The advantages of robotic surgery are more valuable when operating in the confined area, as in the esophageal surgery. The dexterity and articulated instruments permit seven degrees of motion including in/out; rotation; pitch at wrist; yaw at wrist; pitch at fulcrum; yaw at fulcrum and grip strength [20]. The improved tremor free motion stability can add to fine movements and facilitate a precise dissection and suturing in a confined operating space. As a result, we enjoyed atraumatic dissection during the mediastinal dissection of the esophagus and surrounding lymph nodes. Additionally, we did not encounter any iatrogenic trauma during the procedure and achieved advantages in operative time and blood loss.

In comparison to previously reported studies, our study showed reduced total operating time. The total operating time of the procedure in our series was 204.94 mins. The first performed robotic-assisted esophagectomy in 2003 reported total operative time of 246 mins [28]. Subsequent case series by van Hillgers et al. reported total operative time of 450 (range 370–550) min and thoracoscopic time of 180 (range 120–240) mins from experience in 21 patients [9]. Boone et al. reported median operative time of 450 mins in a series of 47 patients [27]. Some of the transhiatal approached had reported total operative time of 267.71 mins [22] and 311 mins [23]. In the report from Sarkaria et al. the median total operative time was 556 min (range 395–807) [24]. The relatively low total operative time in our series was a result of increased experience in robotic surgery, a well focused operating team as well as nursing staff’s familiarity with the procedures and equipments. We were able to reduce the docking time from 30 mins in initial days to 5 mins in the most recent case. This significant decrease represents the learning curve of the surgeon and team. The estimated blood loss was 86.75 ml in our study. Other publications have reported blood loss ranging from 40–625 ml using similar approach [9,21,27]. Papers focused on the transhiatal approach reported blood loss of 54 ml [22] and 97.2 ml [23]. None of our patient required blood transfusion. This is of clinical significance as various studies have indicated that esophageal cancer patients with major blood loss receiving blood transfusions have a worse prognosis [29,30].

The mean ICU stay and hospital stay in our study was 1.2 days and 8 days, respectively. Other studies have reported ICU stay ranging from 1.8 day to 4 days [9,22,27] and hospital stay ranging from 8.7 days to 18 days [9,22-24,27]. Weksler B et al. compared the robotic esophagectomy with the traditional MIE and found no significant differences in operative time, blood loss, number of resected lymph nodes, length of ICU/hospital stay and postoperative complications [31].

In our study, we did not encounter any in-hospital mortality. Approximately, 80% of the patient population was alive at the median follow up of 10 months. There were no treatment related deaths. Two patients had recurrence of the cancer, one of which died while other was disease free following the further treatment. The complication rate was low in our study with reported complications in only 19.28% of the study population. In general, the transthoracic approach is more aggressive than the transhiatal approach and is more likely to cause cardiopulmonary complications, anastomotic and chylous leaks, vocal cord paralysis, and wound infection [32]. However, a study by Satoh et al. has showed significantly reduced incidence of recurrent nerve palsy by robotic thoracoscopic esophagectomy in comparison to conventional thoracoscopic esophagectomy [33]. Although, we encountered only two cases with recurrent nerve palsy, we advocate extreme caution during the en-bloc resection as there are chances of damaging recurrent nerve and its small branches that are located in the fatty tissue of the superior mediastinum. In our series, the post-operating complications reduced markedly in due course, with reported 3 cases anastomotic leak from first 32 cases in our previous publication [11] and no further incidences from last 51 cases. Similarly, there were no further cases of chyle leak from our last 51 cases.

A prospective randomized clinical trial is underway for comparing robot-assisted thoraco-laparoscopic esophagectomy with the open transthoracic esophagectomy [34]. We are really hopeful that the trial will furnish the similar results to our study for robotic esophagectomy. If the trial hypothesis is proved, robot esophagectomy can be considered as treatment option related with a lower postoperative complications, lower blood loss and shorter hospital stay with at least similar oncologic outcomes and better postoperative quality of life [34].

The major drawback of the robotic system is the lack of haptic sensations. This limitation is mainly significant in procedures where touch is an important component. However, the recent surgical innovations are focused on the development of systems that transmits the haptic feedback to surgeon [35]. As the surgeon works alone at a console, learning procedures and training to others can be sometimes challenging [20]. Finally, the cost of the equipments could be additional limitation. However, robot- assisted surgery has already confirmed cost savings from minimal blood loss, morbidity and reduced hospital stay [8].

## Conclusion

We found robot-assisted thoracoscopic esophagectomy acceptable for treatment of esophageal cancer. The procedure allowed precise en-bloc dissection with lymphadenectomy in mediastinum. The advantages of robotic system helped us to minimize operative time, blood loss and complications. We are optimistic that future randomized trials will establish this procedure as standard of care for esophageal cancer.

## Additional file

Additional file 1:
**Robotic Transthoracic Esophagectomy-video.**
